# Tailored TiO_2_ Nanoparticles for Broad-Spectrum Antibiofilm Applications: A Systematic Comparison of Structural and Functional Properties of Carbon- and Nitrogen-Doped TiO_2_ Nanoparticles

**DOI:** 10.1021/acsaenm.5c01089

**Published:** 2026-02-03

**Authors:** Yu Hsin Tsai, Maheshika Kumarihamy, Nicole Beatrice Ponce, Md. Masud Alam, Wooram Kim, Xiong Yu, Tae Kyong John Kim, Anna Cristina S. Samia

**Affiliations:** † Department of Chemistry, 2546 Case Western Reserve University, 10900 Euclid Avenue, Cleveland, Ohio 44106, United States; ‡ Department of Civil and Environmental Engineering, Case Western Reserve University, 10900 Euclid Avenue, Cleveland, Ohio 44106, United States; § Swagelok Center for Surface Analysis of Materials, 2546 Case Western Reserve University, 10900 Euclid Avenue, Cleveland, Ohio 44106, United States

**Keywords:** antibiofilm, antimicrobial photodynamic therapy, nonmetal doping, photocatalysts, titanium dioxide nanoparticles

## Abstract

Nonmetal doping extends the photocatalytic response of TiO_2_ nanoparticles (NPs) into the visible light region; however, systematic evaluations of how specific dopants influence their antimicrobial performance remain limited. In this study, we present a direct comparison of carbon-doped TiO_2_ (C-TiO_2_) and nitrogen-doped TiO_2_ (N-TiO_2_) NPs synthesized via a sol–gel method. Structural and optoelectronic properties were characterized by powder X-ray diffraction (p-XRD), transmission electron microscopy (TEM), attenuated total reflectance Fourier transform infrared spectroscopy (ATR-FTIR), UV–vis diffuse reflectance spectroscopy (UV–vis DRS), and X-ray photoelectron spectroscopy (XPS), confirming dopant incorporation and band gap narrowing. Carbon doping resulted in a more pronounced band gap reduction (2.66 eV compared with 3.09 eV for N-TiO_2_), which correlated with stronger visible light absorption and increased reactive oxygen species (ROS) generation. Under visible light irradiation, C-TiO_2_ NPs achieved 80% eradication of *Staphylococcus aureus* biofilms and 69% eradication of *Escherichia coli* biofilms, corresponding to a ∼1.5-fold higher antibiofilm activity relative to N-TiO_2_ NPs. Differences in bacterial susceptibility were associated with cell envelope architecture, in which the outer phospholipid membrane of Gram-negative *Escherichia coli* likely limited ROS penetration and contributed to lower eradication efficiency compared with Gram-positive *Staphylococcus aureus*. These findings demonstrate that dopant selection directly modulates photocatalytic functionality and identify C-TiO_2_ NPs as a broad-spectrum antimicrobial material. The results have implications for the rational design of TiO_2_-based nanomaterials in antimicrobial photodynamic therapy (aPDT), indoor building environments where pathogen control is essential, environmental remediation, and the development of next-generation self-disinfecting surfaces.

## Graphical abstract

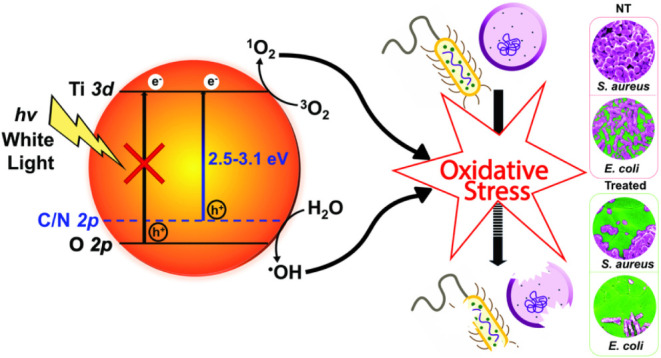

## 1 Introduction

In today’s built environment, maintaining hygienic indoor surfaces is a growing concern across hospitals, transportation hubs, and public infrastructure.^1^ The persistence of bacterial biofilms and resilient microbial consortia that anchor to surfaces and withstand conventional disinfection methods pose serious risks to human health and infrastructure integrity.^2^ As antibiotic resistance continues to rise, engineering durable, light-activated antimicrobial materials has become a critical goal in the design of smart indoor environments.^3^ Surfaces that can autonomously eliminate pathogens using ambient indoor lighting offer a sustainable approach to biofilm control.^4^ However, the challenge lies in developing materials that are not only antimicrobial but also cost-effective, chemically stable, and adaptable for integration into architectural coatings, paints and filtration systems. One strategy that has gained considerable attention is the use of photocatalytic nanomaterials, which can transform passive indoor surfaces into active antimicrobial infrastructures.^5^

Photocatalytic metal oxide nanomaterials generate reactive oxygen species (ROS) under light irradiation that effectively kill bacterial cells and thereby impart long-term antimicrobial properties to surfaces.^6,7^ In particular, semiconductor metal oxides such as TiO_2_, ZnO, and WO_3_ are well-known photocatalysts with demonstrated potential to eradicate resilient bacterial biofilms.^8–10^ Among these, TiO_2_ nanoparticles (NPs) are the most widely investigated, as they combine excellent photoactivity, high chemical stability, low toxicity, and relatively low cost for large-scale deployment compared with other photocatalytic materials.^11^ These properties have enabled the integration of TiO_2_ NPs into various applications including antimicrobial wall coatings and paints for indoor environments, self-sterilizing surfaces in public infrastructure, antimicrobial flooring systems, and antimicrobial textiles used in architectural interiors.^6,12–14^ However, pure TiO_2_ is only activated by ultraviolet (UV) light,^15^ which is not compatible with typical indoor lighting conditions.

Achieving visible light activation requires overcoming the fundamental optical limitations of TiO_2_. Specifically, its intrinsic wide band gap nature (∼3.2 eV) restricts photoactivity to the UV region, thereby limiting practical applications under visible light conditions.^16^ As a result, the photocatalytic activity of TiO_2_ NPs is generally inadequate for the development of broad-spectrum antimicrobial surface coatings.^9^ Indoor lighting conditions, which span the entire visible spectral range and contain only minimal UV irradiance (typically on the order of a few hundred μW cm^–2^, which is lower than outdoor sunlight),^17^ impose strong constraints on the photocatalytic performance of native TiO_2_ under indoor illumination.

To overcome this limitation, different doping strategies have been investigated.^18–25^ Scheme 1 illustrates the band gap narrowing induced by metal and nonmetal doping, which facilitates photoactivation under visible light irradiation compared to intrinsic TiO_2_ NPs. Incorporation of transition metal dopants such as Fe^3+^, Cr^3+^, Cu^2+^, and Ni^2+^ into TiO_2_ NPs has been shown to improve photocatalytic efficiency by introducing defect states within the band structure, thereby enabling photoactivation under white light, as depicted in Scheme 1a.^19^ These dopant-induced defect states typically lie ∼0.5–1.0 eV below the conduction band.^20^ Upon white light irradiation, these states can be populated by photoexcited electrons, a portion of which may subsequently transfer to the Ti 3*d* orbitals, contributing to enhanced photocatalytic activity.^21,22^ Consistent with this mechanism, Karuppasamy et al. reported that transition metal-doped TiO_2_ NPs exhibit reduced effective band gaps, which result in enhanced absorption and improved photocatalytic performance under visible light irradiation compared to pristine TiO_2_.^23^ However, these approaches have not been widely adopted due to poor reproducibility and limited chemical stability of the synthesized photocatalysts. In addition, transition metal doping often promotes charge carrier recombination and lattice distortions, while requiring expensive ion implantation facilities, making metal-doped TiO_2_ NPs impractical for large-scale applications.^24,25^ Prompted by these limitations, nonmetal dopants such as nitrogen (N), carbon (C), and sulfur (S) have been widely explored, offering promising performance due to their comparable size to oxygen, their ability to form metastable deep-level defect states, and their relatively small ionization energies (Scheme 1b). They also present the advantages of low production cost and significant visible light absorption capability.^5,26–28^ However, S-doping often suffers from poor thermal stability and the tendency to form surface sulfate species, limiting its effectiveness compared with N- and C- doping. In contrast, N- and C- doping to TiO_2_ have demonstrated superior visible light absorption and stability while maintaining low production cost.^29^

**Scheme 1:**
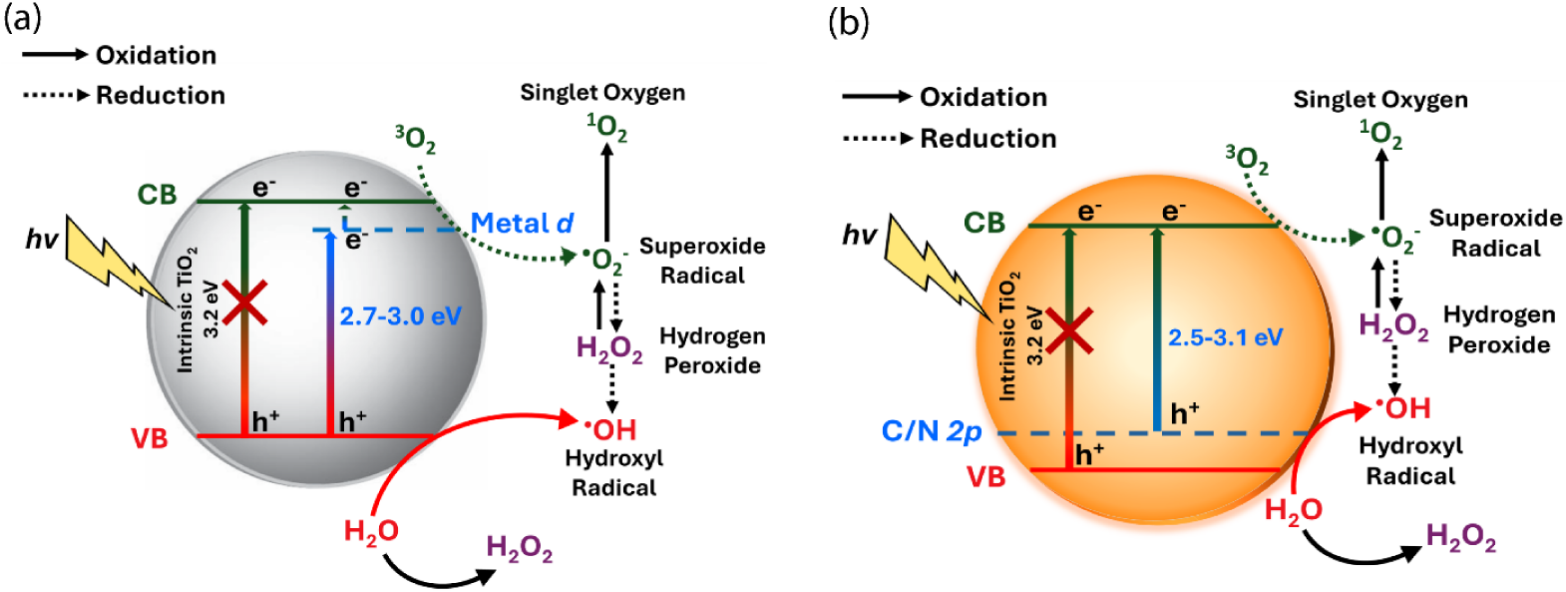
Schematic Representation of Band Gap Narrowing and ROS Generation under Visible Light Excitation[Fn sch1-fn1]

i (a) Band gap narrowing and ROS generation in metal ion doped TiO_2_ NPs. (b) Band gap narrowing and ROS generation in nonmetal doped TiO_2_ NPs.

In this context, a pioneering study by Asahi et al. first demonstrated visible light activity in anatase TiO_2_ doped with N. This activity originated from band gap narrowing caused by the hybridization of the N 2*p* orbitals with the O 2*p* orbitals, forming a delocalized valence band within the N-doped TiO_2_ lattice.^30^ Building on this progress, Burda et al. developed a versatile sol–gel method to synthesize N-doped TiO_2_ (N-TiO_2_) NPs with high nitrogen concentrations, which improved the synthetic accessibility of N-doping.^31^ The electronegativity of the dopant has since been identified as a critical factor in preventing charge recombination during the photocatalytic activation of TiO_2_.^32^ Lower dopant electronegativity facilitates carrier transmission and separation, thereby minimizing charge recombination.^32^ This difference suggests that C-doped TiO_2_ (C-TiO_2_) may outperform N-TiO_2_ in photocatalytic performance. It is proposed that the less electronegative carbon dopant donates greater electron density compared with nitrogen, facilitating improved charge carrier availability and thereby enhancing white light photocatalytic activity.^33^ Additionally, Sakthivel et al. reported that C-TiO_2_ is approximately five times more active than N-TiO_2_ in the degradation of 4-chlorophenol under 445 nm irradiation, supporting this hypothesis.^34,35^ Overall, the enhanced charge separation, favorable ionic radius compatibility, and stronger orbital overlap with oxygen contribute to the superior visible light activation achieved through band gap narrowing by nonmetal dopants.

The photophysical properties of C-TiO_2_ and N-TiO_2_ NPs have been extensively studied for various applications including bacterial biofilm eradication.^36,37^ Despite these individual investigations, the field still lacks systematic comparative studies evaluating their photophysical properties under consistent experimental conditions. In the present work, we address this gap by conducting, for the first time to our knowledge, a detailed side-by-side analysis of C-TiO_2_ and N-TiO_2_ NPs, with particular emphasis on their photocatalytic antimicrobial efficacy against biofilms formed by two clinically relevant bacteria: *Staphylococcus aureus* (*S. aureus*) and *Escherichia coli* (*E. coli*) strains. The first part of this study provides an in-depth comparison of the structural properties between C-TiO_2_ and N-TiO_2_ NPs, focusing on their crystallinity, particle size, surface chemistry, and composition. The second part evaluates the photocatalytic reactive oxygen species (ROS) generation capabilities of the two systems. Finally, a comprehensive assessment is presented to compare the photocatalytic antibiofilm efficacy of C-TiO_2_ and N-TiO_2_ NPs in eradicating *S. aureus* and *E. coli* biofilms, representing Gram-positive and Gram-negative bacterial cell types, respectively. These two bacterial species were selected because Gram-positive and Gram-negative microbes differ markedly in their pathogenic potential and resistance mechanisms against antibiotic therapies—differences that arise from their structural characteristics. Gram-positive bacteria possess a thick, porous peptidoglycan cell wall surrounding the cytoplasmic membrane but lack an outer membrane.^38^ In contrast, Gram-negative bacteria have a complex cell envelope composed of an outer membrane, thin peptidoglycan layer, and an inner cytoplasmic membrane.^39^ Despite these structural differences, both Gram-negative and Gram-positive bacteria are responsible for life-threatening infections that impact both environmental safety and human health.^40,41^ *E. coli* is frequently associated with foodborne public health crises. For example, in 2018, a severe outbreak in the United States linked to romaine lettuce contaminated with *E. coli* *-*containing irrigation water resulted in more than 200 reported infections and serious complications.^42^ In contrast, *S. aureus* remains a major pathogen in healthcare settings. Methicillin-resistant *S. aureus* (MRSA) causes severe hospital-acquired infections, including bloodstream infections and pneumonia.^43^ Therefore, the development of advanced materials for the effective eradication of these bacterial biofilms is of critical and timely importance in addressing these persistent health and environmental threats.

Motivated by these needs, this study aims to provide a comprehensive understanding of the physical, photochemical, and antimicrobial properties of C- and N-doped TiO_2_ NPs. In addition to their biofilm-eradicating potential, these materials hold promise as functional additives in engineering applications such as self-cleaning coatings, antimicrobial polymers, and disinfecting agents. The comparative evaluation presented here is intended to identify the relative strengths and limitations of these two photocatalysts, thereby providing deeper insights into their potential applications in antimicrobial photodynamic therapy (aPDT). Such understanding can guide more informed and targeted utilization of these low cost, high performance photocatalysts in real-world scenarios.

## 2 Materials and Methods

### 2.1 Materials and Reagents

The following chemicals, which were used in the synthesis of the different types of TiO_2_ NPs were obtained from Sigma-Aldrich (St. Louis, MO) and used as-received: titanium tetraisopropoxide (97%), titanium tetrachloride (99%), ethylenediamine (≥99%), 1-hexanol (anhydrous), acetic acid (≥99%), tetrabutylammonium hydroxide solution (1.0 M in H_2_O), ethanol (200 proof), hydrochloric acid (ACS reagent, 37%), and titanium­(IV) oxide—anatase (control undoped sample, 99.7% trace metals basis, <25 nm particle size). The different reactive oxygen species quantification assay kits were obtained from ThermoFisher Scientific (Eugene, OR): hydroxyphenyl fluorescein (HPF, hydroxyl radical sensor), singlet oxygen sensor green (SOSG, singlet oxygen sensor), and 10-acetyl-3,7-dihydroxyphenoxazine (Amplex red, hydrogen peroxide sensor). *Staphylococcus aureus* *(ATCC 49525) and* *Escherichia coli* *(ATCC 25922)* bacterial strains were obtained from American Type Culture Collection (ATCC) (Manassas, VA). Cell culture media components consisting of Luria broth (LB) powder containing 50% tryptone, 25% yeast extract, and 25% sodium chloride, and kanamycin sulfate were purchased from Sigma-Aldrich (Burlington, MA). Additional cell culture supplies and reagents including vitamin K1, fetal bovine serum, crystal violet, paraformaldehyde (4% in PBS), PBS solution (10×), and well plates were all obtained from Fisher Scientific (Pittsburgh, PA). For transmission electron microscopy (TEM), 400-mesh Formvar-coated copper grids were obtained from Electron Microscopy Sciences (Hatfield, PA). For Scanning Electron Microscope (SEM) analysis, sterile Hydroxyapatite (HA) discs (9.7 mm diameter by 1.5 mm thickness) were obtained from Clarkson Chromatography Products (Williamsport, PA).

### 2.2 Synthesis of Nitrogen-Doped TiO_2_ Nanoparticles (N-TiO_2_ NPs)

The N-TiO_2_ NPs were synthesized using a previously reported sol–gel method with minor modifications.^44^ Titanium isopropoxide precursor (13.5 mmol, 4 mL) was mixed with ethylenediamine (0.3 mol, 20 mL) and 1-hexanol (1.2 mol, 150 mL). The resulting reaction mixture was refluxed at 150 °C for 18 h under Ar and then cooled to room temperature. To neutralize excess ethylenediamine before proceeding with the hydrolysis process, 30 mL of acetic acid (≥99%) was added dropwise under constant stirring. The resulting reaction mixture was hydrolyzed by slowly adding 58 mL of deionized water. Following the hydrolysis reaction, a yellow-colored slurry containing the synthesized N-TiO_2_ NPs was obtained. To isolate the N-TiO_2_ NPs, ethanol (200 proof, 15 mL) and deionized water (15 mL) were added to the slurry and the mixture was centrifuged at 7000 rpm for 10 min; this process was repeated twice. The synthesized N-TiO_2_ NPs were dried overnight at 80 °C and ground into a fine powder for further analysis. To remove any remaining organic residues and improve the crystallinity of the material, the prepared N-TiO_2_ powder was annealed at 400 °C for 0.5 h under ambient conditions.

### 2.3 Synthesis of Carbon-Doped TiO_2_ Nanoparticles (C-TiO_2_ NPs)

The C-TiO_2_ NPs were synthesized using a sol–gel method as previously reported.^34^ Briefly, 5 mL of the titanium chloride precursor solution was added dropwise to 50 mL of 0.25 M tetrabutylammonium hydroxide at 4 °C until the pH reached 5.5. After aging the suspension for 24 h at room temperature, the sample was then dialyzed in deionized (DI) water using a dialysis membrane with a 2 kDa molecular weight cutoff to remove reaction byproducts. The DI water was changed approximately five times during this process until a neutral pH was obtained. The purified C-TiO_2_ gel was then dried in an oven overnight at 80 °C, and the synthesized C-TiO_2_ NPs were ground into powder for further analysis. To remove any remaining organic residues and improve the crystallinity of the material, the prepared C-TiO_2_ powder was annealed at 400 °C for 0.5 h under ambient conditions.

### 2.4 Structural, Optical, and Surface Characterization of the Different TiO_2_ NPs

The powder X-ray diffraction (p-XRD) patterns of the control (commercial TiO_2_ anatase) and synthesized TiO_2_ NPs (N-TiO_2_ and C-TiO_2_) were collected using a Rigaku Miniflex 600 powder X-ray diffractometer (Cedar Park, TX) with Cu Kα radiation (λ = 0.154 nm). The diffuse reflectance spectra of the TiO_2_ NP samples were analyzed using a Varian Cary 50 UV–visible spectrophotometer (Cary, NC) with a diffuse reflectance accessory. Attenuated total reflectance Fourier-transform infrared (ATR-FTIR) spectra were collected using a JASCO FT/IR-4600 spectrometer (Oklahoma City, OK). The hydrodynamic diameter and zeta potential of the TiO_2_ NP samples were collected with a Wyatt Mobius dynamic light scattering (DLS)/Zeta potential instrument (Santa Barbara, CA). The elemental composition as well as chemical and electronic states of the atoms within the TiO_2_ NP samples were analyzed using a PHI 5000 Versaprobe X-ray photoelectron spectrometer (XPS) with Al Kα X-ray (1486.6 eV). The photocatalytic experiments were performed using a light illuminator equipped with a white LED head (irradiance at 50 mW cm^–2^, Luzchem Research Inc., Ottawa, Canada). The antibiofilm assays were carried out using a Well Plate illuminator operated at the same irradiance (50 mW cm^–2^, Luzchem Research Inc., Ottawa, Canada).

### 2.5 Quantification of Photogenerated Reactive Oxygen Species (ROS)

The photoactivities of the different TiO_2_ NP samples were evaluated using a light-emitting diode (LED) photoreactor under white LED light (50 mW/cm^2^) illumination for 30 min. Three molecular probes were used to detect specific ROS: HPF for hydroxyl radicals, SOSG for singlet oxygen, and Amplex Red for hydrogen peroxide. For hydroxyl radical detection, 10 μM HPF in DI water with 0.1 mg/mL TiO_2_ NP suspensions were irradiated, and fluorescence signals were recorded at 515 nm with 1 min intervals for the first 10 min, followed by 10 min intervals for a total duration of 30 min. Singlet oxygen detection involved 10 μM SOSG with 0.1 mg/mL TiO_2_ NP suspensions, and fluorescence signals were measured at 525 nm in the same manner. Hydrogen peroxide detection utilized 50 μL of 0.1 mg/mL TiO_2_ NP suspensions in a 24-well plate, which were irradiated, mixed with Amplex Red, and fluorescence signals were read at 575 nm after 30 min of incubation. ROS concentrations were determined using calibration plots from known standards.

### 2.6 Preparation of Bacterial Biofilms for Antibiofilm Assays

*Staphylococcus aureus* (*S. aureus*) was cultured aerobically in Luria Broth containing kanamycin sulfate (200 μg/mL), referred to as LBK, at 37 °C for 24 h. The bacterial cell cultures were diluted to an optical density of 0.7 at 600 nm (OD_600_) and further diluted to a 1:700 ratio using LBK broth, yielding a final concentration of 2 × 10^5^ CFU/mL (OD_600_ = 0.01). Meanwhile, *Escherichia coli* (*E. coli*) was cultured aerobically in Luria Broth without kanamycin at 37 °C for 24 h. The bacterial cell cultures were diluted to an optical density of 0.4 at 600 nm (OD_600_) and further diluted to a 1:400 ratio using LBK broth, yielding a final concentration of 2 × 10^5^ CFU/mL (OD_600_ = 0.01).

### 2.7 Bacterial Biofilm Eradication with the Different Photoactivated TiO_2_ NPs

Bacterial biofilms were cultivated overnight in 24-well cell culture plates at appropriate cell densities using specific growth media for each strain. After biofilm formation, the wells were treated with different TiO_2_ NP suspensions and exposed to white LED light (50 mW/cm^2^ for 30 min) using a Well Plate Illuminator (Luzchem, Ontario, Canada). Following irradiation, the treatments were aspirated, and 500 μL of 0.1% aqueous crystal violet solution was added to each well and incubated for 20 min. Excess crystal violet was aspirated, and the plates were rinsed three times with 1 mL DI water. To quantify the remaining biofilms, the crystal violet stains were dissolved in 1 mL of 30% acetic acid, diluted at a 1:8 ratio, and absorbance values were measured at 595 nm to determine the minimum biofilm eradication concentration (MBEC) for each TiO_2_ NP treatment.

### 2.8 Scanning Electron Microscopy (SEM) Analyses of Biofilm Eradication on Hydroxyapatite Discs

Hydroxyapatite (HA) discs with preformed bacterial biofilms were treated with the different TiO_2_ NP samples and subsequently photoactivated with white LED light. After treatment, the discs were fixed in 4% paraformaldehyde in PBS for 60 min. Fixed samples were rinsed three times with 0.1 M PBS, then progressively dehydrated through a graded ethanol series (50%, 70%, 80%, and 100% ethanol for 10 min each) and dried overnight under vacuum at room temperature. The treated HA discs were sputter-coated with a thin layer of gold and examined using an FEI Quanta 450 FEG environmental SEM at 5 kV to capture images of any residual biofilm structures.

## 3 Results and Discussion

### 3.1 Synthesis and Characterization of C- and N-Doped TiO_2_ NPs

The nonmetal doped TiO_2_ NPs were synthesized using a modified sol–gel method adapted from previously reported protocols, which facilitates direct incorporation of dopant species during the initial hydrolysis and condensation steps.^34,44^ Specifically, the N-TiO_2_ NPs were prepared using ethylenediamine as the nitrogen source during the hydrolysis of titanium isopropoxide under reflux.^44,45^ Controlled pH adjustment and hydrolysis were achieved through dropwise addition of acetic acid and water, respectively, under vigorous stirring. This reaction environment serves two key functions: (i) the amine groups of ethylenediamine act as nitrogen donors that coordinate to Ti^4+^ ion centers through Ti–N linkages, and (ii) the moderate hydrolysis rate prevents premature condensation of the titania network, thereby promoting homogeneous dopant incorporation throughout the sol–gel matrix.^46^ The resulting yellow titania gel is amorphous; to promote crystallization and remove residual organic species, the isolated NPs were annealed at 400 °C for 0.5 h.^46^ In contrast, the C-TiO_2_ NPs were prepared by the alkaline hydrolysis of titanium chloride in the presence of tetrabutylammonium hydroxide (TBAOH), as C source.^34,47^ Uniform nucleation and prevention of premature aggregation of the titania sol clusters were achieved by the gradual addition of TiCl_4_ into the TBAOH solution until the pH reached 5.5.^47,48^ When TiCl_4_ is added to the aqueous TBAOH solution, Ti^4+^ ions undergo controlled hydrolysis, forming Ti–OH and Ti–O–Ti linkages while liberating HCl. The presence of this acidic byproduct lowers the pH and can lead to reprotonation of the formed Ti–OH groups, destabilizing the gel matrix and hindering dopant incorporation. To remove HCl and other byproducts, the as-formed gel was dialyzed against DI water through a 2 kDa membrane. The repeated exchange of DI water (≈5 cycles) enabled diffusion-driven removal of free H^+^ and Cl^–^ ions, gradually restoring the pH to neutral and leaving behind a purified Ti–O–Ti sol–gel network containing uniformly distributed carbon precursors from TBAOH. The resulting white titania gel is amorphous and to promote crystallization and remove residual organic species, the isolated NPs were annealed under similar conditions as for the N-TiO_2_ NPs.^34^ Both synthesis routes resulted in nanocrystalline TiO_2_ with distinct dopant induced optoelectronic properties.

To investigate the optical properties of the synthesized TiO_2_ NP samples, diffuse reflectance spectroscopy (DRS) analyses techniques were performed using a UV–visible spectrophotometer (Figures 1a and S1) equipped with an integrating sphere attachment, which enables accurate collection of diffusely reflected light from the powdered samples. The inset of Figures 1a and S2 show the physical appearance of the as-synthesized NPs. The results indicated that both N-TiO_2_ and C-TiO_2_ NP samples exhibited enhanced absorbance, particularly in the 400–600 nm visible light range, confirming successful band gap narrowing through nonmetal doping. The annealed N-TiO_2_ and C-TiO_2_ samples appeared orange in color, consistent with their enhanced visible-light absorption. The orange hue originates from selective absorption in the blue-green spectral region (≈450–550 nm), resulting in reflected and transmitted light dominated by longer red-orange wavelengths. This optical response is characteristic of dopant-induced midgap energy states within the TiO_2_ lattice, which facilitates sub-band gap electronic transitions and thereby extend the material’s photoresponse into the visible spectrum.^33^

**Figure 1:**
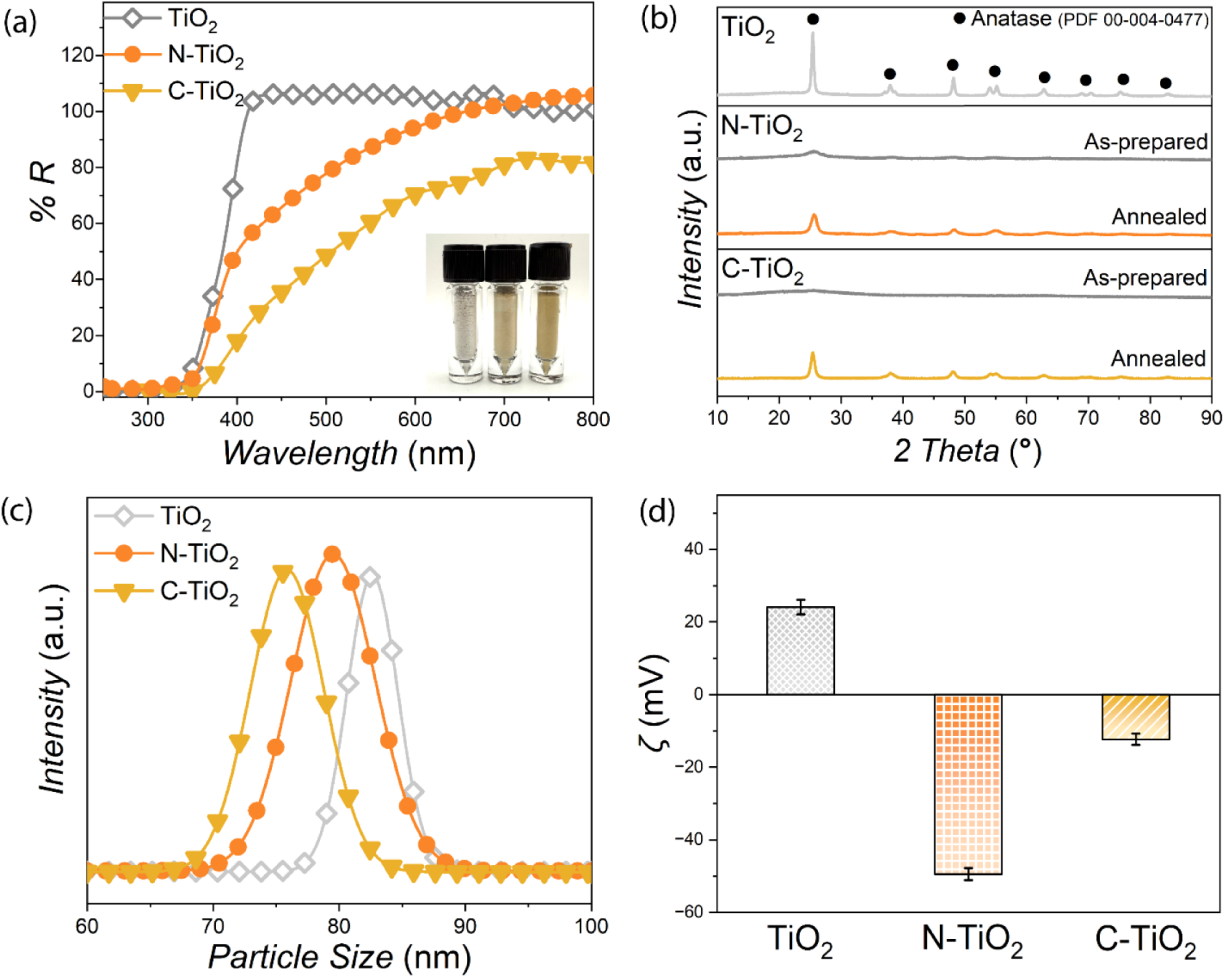
(a) Diffuse reflectance (DR) spectra, (b) powder X-ray diffraction (p-XRD) patterns, (c) dynamic light scattering (DLS) spectra, and (d) zeta potential data collected from the different TiO_2_ NP samples: commercial titania NPs (TiO_2_ NPs), N-doped titania NPs (N-TiO_2_ NPs), and C-doped titania (C-TiO_2_ NPs), respectively. The X-ray diffraction reference for the anatase titania phase (PDF-00-004-0477) was adapted from the International Centre for Diffraction Data (ICDD) database.

The optical band gap energies of the synthesized NPs were estimated using Tauc plot analysis (Figure S3) derived from the Kubelka–Munk function applied to the diffuse reflectance spectra.^49,50^ The Kubelka–Munk function (eq 1), where *R*_∞_ is the reflectance of an infinitely thick sample, converts reflectance data into an equivalent absorption coefficient.^50^ According to the Tauc relation (eq 2), the optical band gap (*E*_g_) can be determined by plotting (αhν)^*n*^ against hν, where α is the absorption coefficient, hν is the photon energy, *A* is a proportionality constant, and *n* characterizes the nature of the electronic transition taken as 1/2 for indirect allowed transitions typical of anatase TiO_2_. By plotting (αhν)^1/2^ against hν and extrapolating the linear portion of the curve to the photon energy axis, the intercept yields the optical band gap energy to TiO_2_ based systems.^50,51^

1 $$f\left(R_{\infty }\right)=\frac{{\left(1-R_{\infty }\right)}^{2}}{2R_{\infty }}$$

2 $${\left(\alpha h\nu \right)}^{n}=A\left({h\nu -E}_{g}\right)$$

The Tauc plots shown in Figure S3 revealed that the undoped TiO_2_ sample exhibited a band gap of 3.16 eV, consistent with pristine anatase TiO_2_ (≈3.1–3.2 eV).^50^ The N-TiO_2_ and C-TiO_2_ samples showed band gap narrowing to 3.09 and 2.66 eV, respectively, confirming the introduction of dopant-induced midgap states that extend the absorption edge into the visible region. Similar trends have been reported in previous studies, where nitrogen doping induces N *2p* states near the valence band, producing modest red shifts (≈3.0–3.1 eV).^33^ In contrast, carbon doping can lead to more pronounced band gap narrowing (≈2.6–2.8 eV) due to substitutional C–Ti bonding and interstitial C–O–Ti configurations introducing electronic states deeper within the band gap.^52^ These results therefore align well with the previously reported optical behavior of C- and N-doped TiO_2_ NPs, confirming successful dopant incorporation and visible light photoactivation.

The p-XRD patterns collected for the different TiO_2_ NP samples highlight the transition from an amorphous to crystalline structure following postsynthesis heat treatment at 400 °C for 0.5 h in ambient air (Figure 1b). After annealing, both C-TiO_2_ and N-TiO_2_ NP samples exhibited diffraction peaks characteristic of the anatase crystalline phase, confirming successful crystallization. The heat treatment also served to remove residual organic species, as verified by attenuated total reflectance ATR-FTIR analyses (Figure S4). Figure S4 shows the ATR-FTIR spectra obtained for the different TiO_2_ NP samples. The as-synthesized N-TiO_2_ NPs exhibited functional groups characteristic of the organic precursors used during synthesis, while the as-synthesized C-TiO_2_ NPs displayed IR vibration bands associated with tetrabutylammonium hydroxide, consistent with the carbon doping precursor. Notably, the intensity of these organic surface functional groups both in N- and C-TiO_2_ NP samples decreased markedly after heat treatment at 400 °C for 0.5 h under ambient air conditions, confirming the successful removal of adsorbed residual organic precursors and byproducts during the annealing process.

TEM analysis was conducted to examine the morphology and particle size distribution of the synthesized TiO_2_ NPs (Figure S5). Both C-TiO_2_ and N-TiO_2_ NP samples exhibited predominantly spherical morphologies with narrow particle size distributions. The average particle size was estimated to fall within the 15–25 nm range, in good agreement with previously reported sol–gel derived TiO_2_ NPs.^5,52^

The average hydrodynamic diameters of the different TiO_2_ NP samples were characterized using DLS (Figure 1c). The measured hydrodynamic sizes for TiO_2_, N-TiO_2_, and C-TiO_2_ NP samples were 82.6 ± 2.5 nm, 79.5 ± 6.8 nm, and 75.9 ± 5.9 nm, respectively. Although the TEM images reveal particle clustering (Figure S5), the narrow, monomodal DLS size distributions indicate comparable hydrodynamic radii across all samples (Figure 1c), confirming the absence of extensive aggregation in suspension and thereby supporting the direct comparison of their antibacterial activities. As expected, the hydrodynamic diameters measured by DLS are larger than the average core sizes observed by TEM due to solvation layers associated with the dispersed NPs. Surface charge properties were evaluated by zeta potential measurements at pH 6.5 (Figure 1d). The commercial TiO_2_ NP sample exhibited a positive surface potential of +24.1 mV, indicative of its pristine state. In contrast, the N-TiO_2_ and C-TiO_2_ NPs displayed negative surface potentials of −49.5 mV and −12.3 mV, respectively, indicating dopant-induced alterations to surface charge states. These shifts are consistent with prior reports on nonmetal doped TiO_2_ systems.^53,54^ For example, increasing nitrogen incorporation has been shown to generate more negative zeta potentials due to substitutional or interstitial N species altering surface charge states.^55^ More broadly, the introduction of heteroatoms can modify the isoelectric point and surface charge distribution of TiO_2_ colloids, often leading to more negative potentials that enhance electrostatic stabilization in aqueous suspensions.^56^ Accordingly, the more negative potential of N-TiO_2_ suggests a stronger influence of nitrogen doping in introducing negatively charged surface sites or suppressing surface hydroxyl protonation under these conditions.^56^ These surface charge differences may influence particle dispersion stability, aggregation tendencies, and ultimately interfacial interactions with substrates or reactants in photocatalytic reactions.^55,57^

The atomic composition of the various TiO_2_ NP samples was analyzed using X-ray photoelectron spectroscopy (XPS) (Figure 2a,b). The appearance of distinct C *1s* and N *1s* peaks in the survey scans, compared with the commercial TiO_2_ (Figure S6), confirms the incorporation of carbon and nitrogen in the synthesized TiO_2_ NPs. Following heat treatment, the carbon content was significantly reduced in both the as-prepared N-TiO_2_ and C-TiO_2_ NP samples, indicating efficient removal of residual organic precursors. Specifically, the as-prepared N-TiO_2_ NP sample showed an initial carbon content of 57.7%, which decreased to 14.7% after annealing. In the C-TiO_2_ NP sample, the carbon concentration decreased from 41.2% to 14.1%, further evidencing the elimination of residual organic species. Additionally, the nitrogen content in the N-TiO_2_ NP sample decreased from 18.9% to 5.5% upon annealing, indicating that a portion of nitrogen species remained incorporated in the titania crystal lattice. Furthermore, the Cl *2p* signal from the as-prepared C-TiO_2_ NP sample (initially 7.4%, originating from the TiCl_4_ precursor) was significantly removed after heat treatment, confirming the effectiveness of the postsynthesis annealing process to pyrolyze adsorbed precursor molecules. Table S1 summarizes the full elemental compositions obtained from the XPS survey spectra for all the TiO_2_ NP samples.

**Figure 2:**
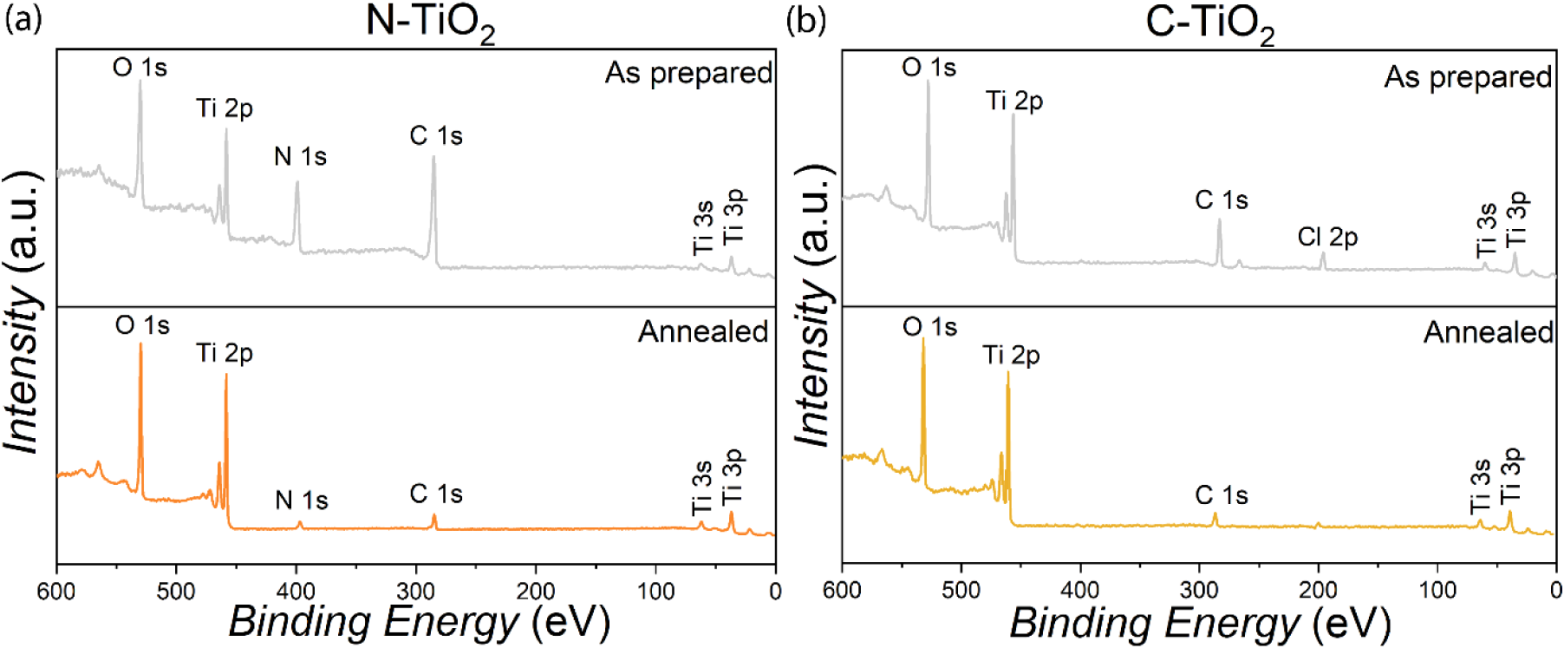
XPS survey scans of (a) as-prepared and annealed N-TiO_2_ NP samples, and (b) as-prepared and annealed C-TiO_2_ NP samples.

High-resolution XPS (HR-XPS) was employed to further investigate the chemical states of the various TiO_2_ NP samples before and after heat treatments. The HR-XPS Ti *2p* spectra (Figure 3a) revealed the presence of both Ti^4+^ and Ti^3+^ species in the N-TiO_2_ NP samples, consistent with nitrogen incorporation inducing partial reduction of Ti centers. The appearance of Ti^3+^ suggests the formation of oxygen vacancies and dopant-induced defect states, which are known to enhance visible light absorption and promote charge carrier separation. In contrast, the commercial TiO_2_ and C-TiO_2_ NP samples exhibited only the Ti^4+^ peak, indicating the absence of detectable Ti^3+^ species under the measurement conditions.

**Figure 3:**
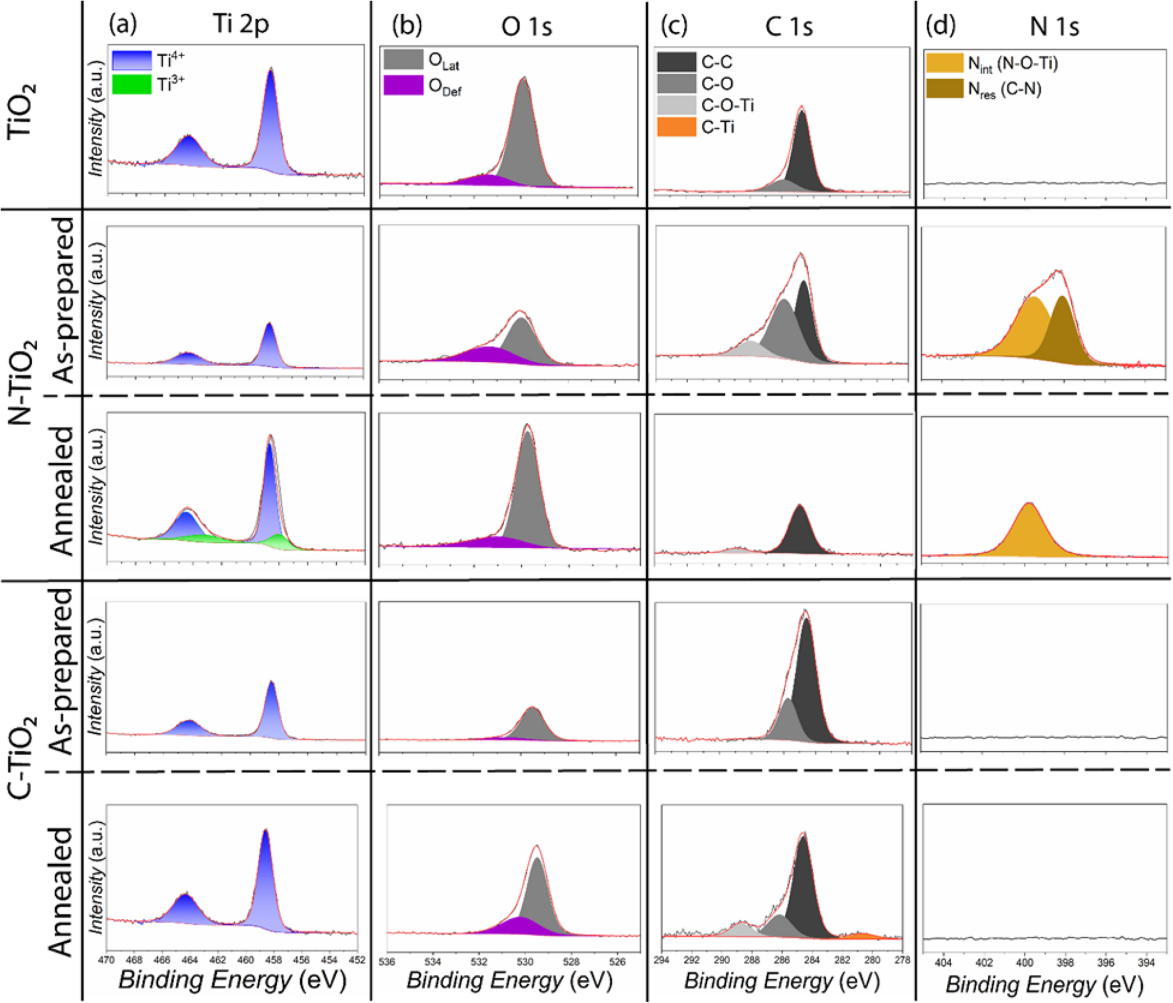
High resolution-XPS spectra of (a) Ti *2p*, (b) O *1s*, (c) C *1s*, and (d) N *1s* collected from commercial TiO_2_, as-prepared N-TiO_2_, annealed N-TiO_2_, as-prepared C-TiO_2_, and annealed C-TiO_2_ NP samples.

In the O *1s* spectra, both the as-prepared N-TiO_2_ and C-TiO_2_ NP samples exhibited broadened and low-intensity features, which can be attributed to significant surface coverage by adsorbed organic residues remaining from the sol–gel synthesis process (Figure 3b). After heat treatment, these impurity-related contributions diminished, revealing distinct lattice oxygen (*O*_Lat_) and defective oxygen (*O*_Def_) components at 529.8 and 530.1 eV, respectively.^58,59^ The presence of both *O*_Lat_ and *O*_Def_ peaks, also observed in the commercial TiO_2_ NP sample, confirms that postsynthesis annealing effectively removed most organics while preserving oxygen vacancy related defect sites beneficial for photocatalytic activity in the visible spectral range.

Similarly, the C *1s* spectra of the as-prepared N-TiO_2_ NP and C-TiO_2_ NP samples primarily reflected organic residues from the sol–gel synthesis precursor molecules (Figure 3c). After heat treatment, the carbon signal substantially decreased, indicating successful removal of these residues. In contrast, the as-prepared C-TiO_2_ NP samples displayed two notable peaks after annealing at 288.0 and 281.0 eV, corresponding to interstitial (C–O–Ti) and substitutional (C–Ti) carbon dopants within the TiO_2_ crystal lattice.^36,60–62^ These results verify that carbon atoms were incorporated as lattice dopants rather than merely adsorbed species. The Cl *2p* spectra of the as-prepared C-TiO_2_ NP sample exhibited distinct peaks of Cl *2p*_1/2_ and Cl *2p*_3/2_, originating from the TiCl_4_ precursor used during hydrolysis (Figure S7).^63^ After annealing, these chlorine peaks completely disappeared, confirming the full removal of chlorine-based residues.

In the N *1s* spectra, the annealed N-TiO_2_ NP sample exhibited a dominant peak at 400.0 eV (*N*_int_), which can be assigned to interstitially bonded nitrogen species (Figure 3d).^37^ Prior to annealing, an additional peak at 398.0 eV (*N*_res_) was observed, indicative of titanium oxynitride species occupying an intermediate state between substitutional and interstitial sites.^64,65^ This intermediate feature disappeared after heat treatment, suggesting that nitrogen dopants preferentially migrated to interstitial sites within the TiO_2_ crystal lattice upon annealing. No detectable N *1s* signal was observed in the C-TiO_2_ NPs, confirming the absence of nitrogen incorporation during synthesis. This observation is consistent with the precursor chemistry, as the TiCl_4_-TBAOH route lacks any nitrogen-bearing reagents, and the oxidative annealing at 400 °C in air would remove any transient amine residues as volatile NO_*x*_ species.

### 3.2 Measurement of the Reactive Oxygen Species (ROS)

The ROS generation measurements were performed for all the TiO_2_ NP samples under 30 min white LED irradiation, using three distinct fluorescent molecular probes, each targeting a specific ROS: HPF for hydroxyl radicals (•OH), SOSG for singlet oxygen (^1^O_2_), and Amplex Red for hydrogen peroxide (H_2_O_2_) (Figure 4). The results revealed a substantial increase in ROS production for C-TiO_2_ relative to N-TiO_2_ across all species, indicating higher photocatalytic efficiency under visible light excitation. This enhanced activity can be attributed to the stronger visible light absorption and greater band gap narrowing in C-TiO_2_, which together promote more efficient photoexcitation and charge-carrier utilization.

**Figure 4:**
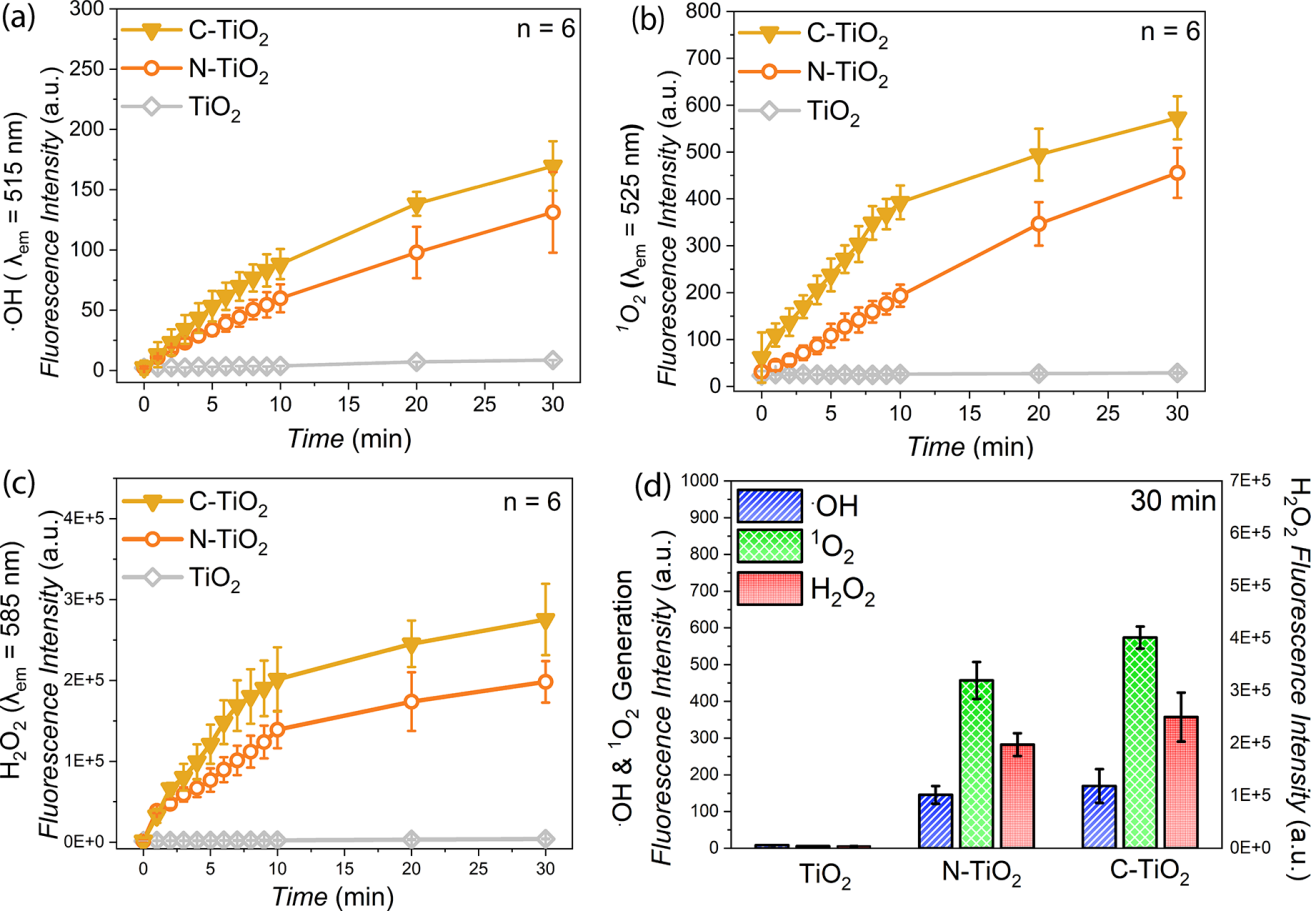
ROS generation from the different TiO_2_ NP samples evaluated under white LED light irradiation (50 mW/cm^2^) for 30 min. The corresponding time-dependent plots show the measured afterglow fluorescence intensity of molecular probes used to detect: (a) hydroxyl radicals (•OH), (b) singlet oxygen (^1^O_2_), and (c) hydrogen peroxide (H_2_O_2_). (d) Fluorescence intensities of the respective ROS molecular probes after cumulative 30 min exposure to white LED light. Error bars represent standard deviation of *n* = 6 replicates. Statistical significance between the ROS generation of N-TiO_2_ and C-TiO_2_ NP samples was evaluated using one-way ANOVA, with p-values annotated.

To ensure reliable detection of photoinduced ROS and to minimize potential probe-NP interactions, all ROS measurements were conducted using established, previously reported methods for similar TiO_2_-based nanomaterials.^66,67^ The ROS probe molecules were separately incubated with each TiO_2_ NP formulation under dark conditions, and measurements at 0 min irradiation were performed in three independent replicates to establish dark measurement baseline. These controls showed negligible signal, indicating the absence of intrinsic or self-generated ROS in the absence of light. In addition, low NP concentrations were employed to minimize aggregation-related effects and potential probe interference. The observed ROS generation was strictly light-dependent, consistent with the photoactivation mechanism of TiO_2_-based photocatalysts and further supported by the lack of antimicrobial activity under dark conditions.

For hydroxyl radical (•OH) generation, C-TiO_2_ exhibited a gradual and sustained increase throughout the 30 min irradiation period, producing ∼16.7% more •OH than N-TiO_2_ (Figure 4a). This incremental yet consistent increase reflects the sustained photocatalytic activity of C-TiO_2_, likely due to its favorable band structure, which facilitates efficient electron–hole separation and ROS generation. Similarly, singlet oxygen (^1^O_2_) production was markedly higher for C-TiO_2_, reaching ∼25.6% higher ^1^O_2_ levels compared to N-TiO_2_ after 30 min light excitation (Figure 4b). Since ^1^O_2_ plays a key role in oxidative stress-mediated photocatalytic processes, these results further highlight favorable electronic states introduced by carbon doping for ROS related photocatalytic applications.

The most pronounced enhancement was observed in hydrogen peroxide (H_2_O_2_) production, where C-TiO_2_ NPs generated ∼26.6% more H_2_O_2_ than N-TiO_2_ by the end of the 30 min irradiation period (Figure 4c). The elevated H_2_O_2_ output is indicative of both enhanced oxygen reduction pathways and suppressed electron–hole recombination in the C-doped lattice. In contrast, commercial TiO_2_ NPs remained inactive toward ROS formation under visible light exposure, as the incident photons were of insufficient energy to excite charge carriers across its wide band gap. This stark performance difference underscores the necessity of nonmetal doping strategies to unlock visible light photocatalytic functionality of TiO_2_-based nanomaterials.

The comparative analysis of ROS formation between the three different TiO_2_-based nanoparticles (Figure 4d) clearly reveals the impact of the two different nonmetal dopants on their photoinduced redox behavior. Upon 30 min of illumination, both N-TiO_2_ and C-TiO_2_ exhibit markedly enhanced generation of •OH, ^1^O_2_, and H_2_O_2_ compared to undoped TiO_2_, signifying improved charge-carrier utilization for oxidative and reductive pathways. Among the doped systems, C-TiO_2_ shows the highest •OH, ^1^O_2_ and H_2_O_2_ yields, whereas N-TiO_2_ displays relatively lower ROS production, consistent with their distinct dopant-induced electronic structures. The pronounced ROS generation efficiency of the doped nanoparticles arises from the presence of Ti^3+^ centers and oxygen vacancies that facilitate electron–hole separation and surface redox reactions. Overall, the superior ROS yield of C-TiO_2_ highlights its more efficient charge-transfer dynamics and defect-mediated redox behavior, establishing it as a more efficient photocatalyst compared to N-TiO_2_ under identical conditions.

### 3.3 Antimicrobial Biofilm Assays

In the context of ROS-mediated oxidative stress on bacterial cell membranes, Gram-positive bacteria are generally more susceptible due to key structural differences compared to Gram-negative species (Scheme 2). Gram-positive bacteria possess a single, thick peptidoglycan layer but lack the additional outer membrane (OM) found in Gram-negative bacteria.^38^ The absence of this OM leaves their cell wall directly exposed to oxidative attack from external agents. The peptidoglycan layer, a rigid polymer composed of alternating *N*-acetylglucosamine (GlcNAc) and *N*-acetylmuramic acid (MurNAc) units cross-linked by short peptide bridges, forms a mechanically strong but chemically penetrable barrier.^68^ Without the protective OM, NPs and ROS can more readily access and oxidize surface structures, initiating lipid peroxidation, protein denaturation, and disruption of membrane integrity.^69,70^ In addition, oxidative destabilization of teichoic acids, which help maintain cell wall rigidity and ionic balance, further increase permeability.^71^ Although Gram-positive bacteria produce antioxidant enzymes as a defense mechanism, their effectiveness is limited compared to the dual-layer protection of Gram-negative bacteria. Prolonged oxidative conditions reduce the efficacy of these enzymes, leading to accelerated cell damage over time.^35^

**Scheme 2:**
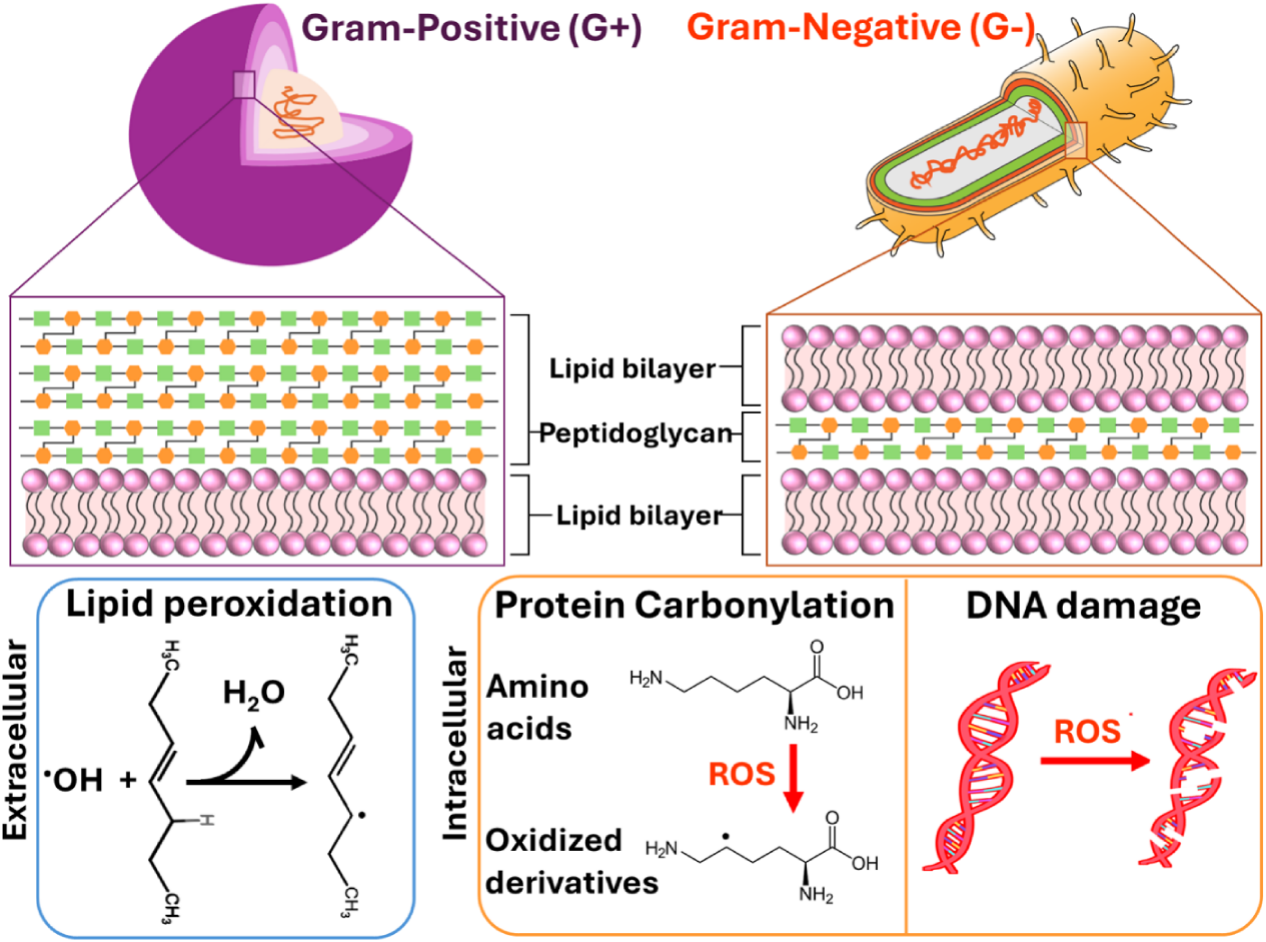
Schematic Illustration of ROS-Induced Damage in Gram-Positive (G^+^) and Gram-Negative (G^–^) Bacterial Cells

In contrast, Gram-negative bacteria benefit from their OM, which provides enhanced resistance to external stressors, including ROS. The OM is composed of lipopolysaccharides (LPS) and embedded proteins that ensure structural integrity and selective permeability, shielding the periplasmic space and cell membrane from oxidative agents.^72^ This protective layer makes Gram-negative bacteria more resilient to ROS-mediated damage. However, high concentrations of ROS can eventually penetrate the OM, triggering lipid peroxidation in the inner lipid membrane.^73^ This process disrupts membrane fluidity, leading to destabilization and cell death. Nonetheless, extreme oxidative stress can overwhelm these defenses, compromising bacterial viability.

Different types of ROS play a critical role in bacterial eradication by targeting both extracellular and intracellular components as shown in Scheme 2. Extracellularly, ROS generated by C-TiO_2_ and N-TiO_2_ disrupt bacterial cell walls through lipid peroxidation. In Gram-positive *S. aureus*, •OH and^1^O_2_ oxidize the peptidoglycan layer, destabilizing the thick network of polysaccharides and peptides. In Gram-negative *E. coli*, ROS primarily attack the LPS layer and phospholipid bilayer, initiating lipid peroxidation that damages the cell membrane. This lipid peroxidation compromises the structural integrity of the membrane, resulting in the leakage of vital ions such as potassium and ultimately reducing bacterial viability.^74–76^

Intracellularly, ROS penetrate through damaged membranes and further target critical cellular components.^1^O_2_ and H_2_O_2_ oxidize cytoplasmic enzymes and metabolic substrates, disrupting essential cellular functions. Furthermore, ROS interacts with the membrane proteins leading to protein carbonylation, which disrupts the membrane transport by impairing the function of channel proteins. Therefore, this process amplifies the damage to other intracellular structures. Additionally, •OH cause oxidative damage to nucleic acids, inducing strand breaks and base modifications that hinder replication and transcription processes, ultimately leading to cell death. The OM of Gram-negative bacteria provide additional protection, making *E. coli* more resistant to intracellular ROS attacks compared to *S. aureus*.^75,76^

To assess the efficacy of the synthesis visible-light activated N- and C-TiO_2_ NP samples in disrupting bacterial biofilms, 1-day-old biofilms of *S. aureus* (Gram-positive) and *E. coli* (Gram-negative) were exposed to white LED light for 30 min in the presence of suspensions of different TiO_2_ NP samples. The minimum biofilm eradication concentration (MBEC) for each sample was determined using a crystal violet staining assay (Figure 5). Under dark conditions, all TiO_2_ NP samples were inactive. However, following 30 min of white LED irradiation, both N-TiO_2_ and C-TiO_2_ NP samples displayed significant antibiofilm activity. C-TiO_2_ NP samples eradicated 80% of *S. aureus* biofilms and 69% of *E. coli* biofilms, while N-TiO_2_ NP samples achieved 55% and 45% biofilm removal for *S. aureus* and *E. coli*, respectively.

**Figure 5:**
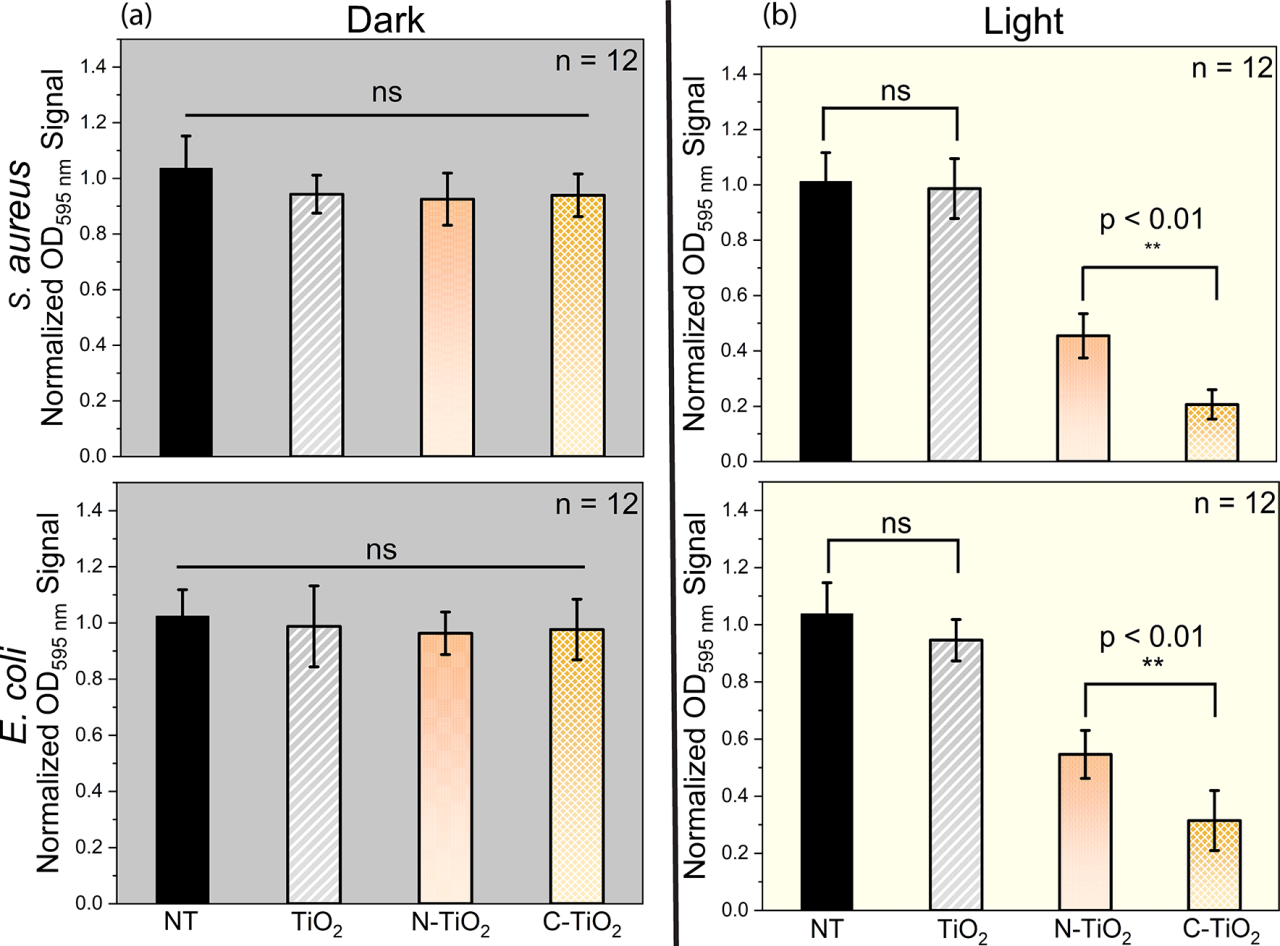
Antibiofilm activity of the different TiO_2_ NP samples (0.1 mg/mL) evaluated by measuring the optical density (OD) of crystal violet stained-biofilms formed by (a) *S. aureus* and (b) *E. coli*. The samples were treated under both dark and light conditions for 30 min. Error bars represent standard deviation of *n* = 12 replicates. Statistical significance was assessed using one-way ANOVA, with p-values annotated.

These results indicate that Gram-positive was generally easier to eradicate than Gram-negative bacteria. This disparity can be attributed to the structural differences between the two types of bacteria. *E. coli* has an additional outer LPS layer that provides extra protection, making it more resistant to antimicrobial treatments. In contrast, the simpler structure of Gram-positive *S. aureus*, with its thick but penetrable peptidoglycan layer, allows for easier ROS-mediated damage.

Figure 6 presents SEM images that illustrate the biofilm coverage of *S. aureus* and *E. coli* grown for 24 h on HA discs, which were then exposed to different TiO_2_ NP treatments under white LED light for 30 min. For *S. aureus*, the NT showed a high biofilm surface coverage of 97.7%, while treatment with commercial TiO_2_ NPs under visible light showed the coverage of 96.5%, indicating no antibiofilm activity. However, N-TiO_2_ NPs showed a significant reduction in biofilm coverage to 49.5%, and C-TiO_2_ NPs further reduced it to 22.7%, suggesting C-TiO_2_ proposed a much higher antibiofilm efficacy.

**Figure 6:**
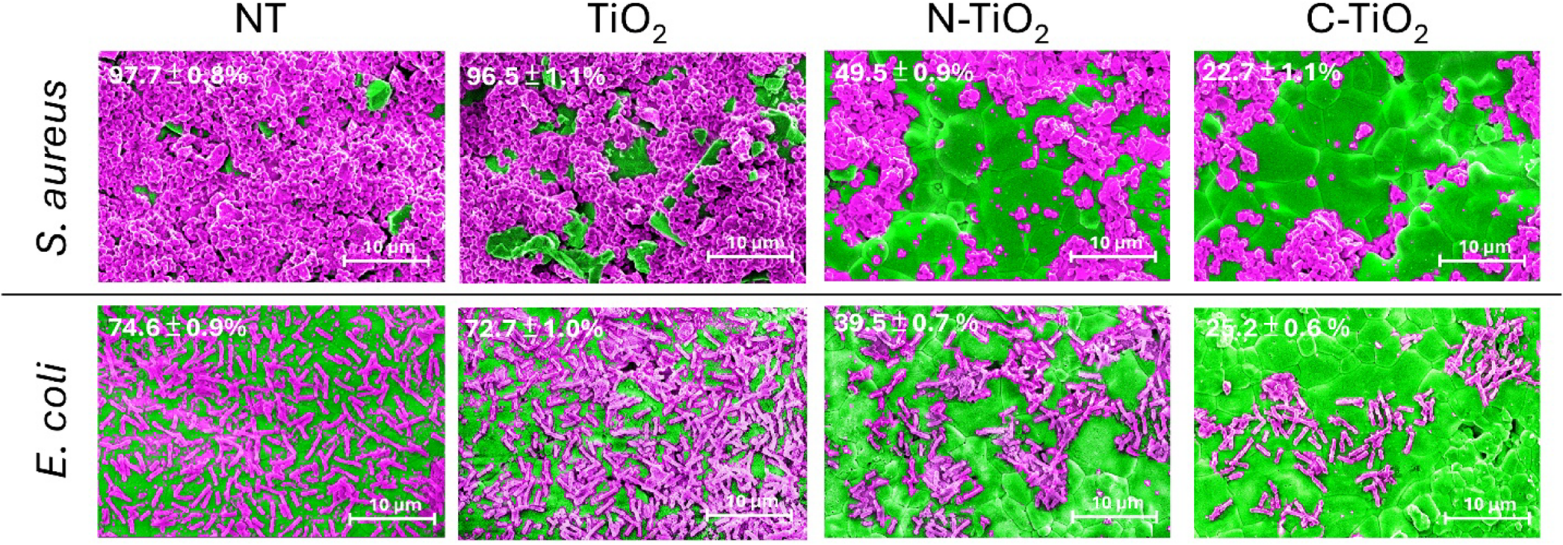
SEM images showing the biofilm coverage of 1-day-old *S. aureus* and *E. coli* on hydroxyapatite (HA) discs. The biofilms were exposed to different treatments under white LED light irradiation for 30 min: untreated control biofilms (NT), biofilms treated with TiO_2_ NPs, biofilm treated with N-TiO_2_ NPs, and biofilms treated with C-TiO_2_ NPs. The insets in the SEM images display the average remaining biofilm coverage after treatment with the different TiO_2_ NP samples (*n* = 3). Biofilm-covered areas are highlighted in purple, and the exposed HA disc surface is colored green to enhance visual contrast. The scale bars represent 10 μm.

For *E. coli*, the NT biofilm sample displayed surface coverage of 74.6%. Commercial TiO_2_ NPs had a negligible effect, reducing coverage only to 72.7%. Treatment with N-TiO_2_ NPs reduced *E. coli* biofilm coverage to 39.5%, while C-TiO_2_ NPs achieved a further decrease to 25.2%. These SEM results align closely with the MBEC assay findings, which similarly showed that C-TiO_2_ had the highest antibiofilm activity against both bacteria. The SEM images confirm that *S. aureus* biofilms were more easily eradicated than *E. coli* biofilms, supporting the trend observed in the MBEC assay. This difference reflects the inherent resistance of Gram-negative bacteria like *E. coli*, which possess an outer membrane that acts as an additional barrier against antimicrobial agents. Moreover, relatively positive surface charge of C-TiO_2_ likely enhances its interaction with the negatively charged bacterial cell walls facilitating greater biofilm disruption. This synergy between electrostatic interactions and photocatalytic activity underscores C-TiO_2_ effectiveness and correlates well with the MBEC results, where C-TiO_2_ consistently outperformed N-TiO_2_ in reducing biofilm coverage across both bacterial species.

Table 1 provides a comparative summary of the antibiofilm performance of the TiO_2_-based NPs investigated in this study alongside representative literature reports. Despite variations in experimental conditions, light sources, and assay formats across different studies, the comparison highlights that the carbon- and nitrogen-doped TiO_2_ NPs evaluated here exhibit antibiofilm efficiencies that are comparable to or exceed many previously reported TiO_2_-based systems, particularly under visible light irradiation. This comparison underscores the effectiveness of dopant-induced band gap modification in enhancing the functional antimicrobial performance of TiO_2_ nanomaterials.

**Table 1:** Comparative Summary of Antibiofilm Performance of Carbon- and Nitrogen-Doped TiO_2_ NPs Investigated in this Study and Representative TiO_2_-Based Systems Reported in Literature

Material	Bacterial Cell	Microbial Assay	Light Excitation	Reported Eradication Outcome	References
C-TiO_2_ NPs	*S. aureus*	Crystal violet	White LED, 50 mW/cm,^2^ 30 min	80%	This study
N-TiO_2_ NPs				55%	
C-TiO_2_ NPs	*E. coli*			69%	This study
N-TiO_2_ NPs				45%	
Epoxy/TiO_2_	*S. aureus*	Crystal violet	UV irradiation, 18 h	43%	Santhosh & Natarajan^77^
Epoxy/Ag-TiO_2_ coatings				67%	
Epoxy/TiO_2_	*E. coli*			56%	
Epoxy/Ag-TiO_2_ coatings				77%	
Green TiO_2_ NPs	*S. aureus*	Crystal violet		80%	Bano et al.^78^
	*E. coli*			89%	
N-TiO_2_ coated surfaces	*S. aureus*	CFU counting	3700 lx, fluorescent light, 80 min	99%	Caratto et al.^79^
	*P. aeruginosa*		3700 lx, fluorescent light, 5 min	77%	
N-TiO_2_ NPs	*S. aureus*	CFU counting	18 W visible lamps, 360 min	100%	Ananpattarachai et al.^80^
N-TiO_2_ NPs	*E. coli*		18 W visible lamps, 420 min	100%	
Ni-TiO_2_ NPs			18 W visible lamps, 300 min	15.3%	
Undoped TiO_2_ NPs				10.5%	
N-TiO_2_ thin films	*E. coli*	CFU counting (film)	3 × 10^4^ lux, incandescent lamp, 5–25 min	80–95%	Wong et al.^81^
N-TiO_2_ NPs	*S. aureus*	Crystal violet	0.2 W cm^–2^, 405 nm LED, 3 h	57.5%	Chen et al.^82^
Undoped TiO_2_ NPs				14.2%	
C-TiO_2_ nanoflakes (RGO-TNFs)	*S. aureus*	CFU counting	Natural sunlight, 40 min	85%	Ghumro et al.^27^
	*E. coli*			80%	

## 4 Conclusion

In this study, we demonstrated the enhanced photocatalytic efficiency and antibiofilm activity of TiO_2_ NPs achieved through nonmetal doping. Among the doped samples, C-TiO_2_ NPs exhibited superior performance compared to N-TiO_2_, primarily due to the more effective band gap narrowing introduced by carbon doping. This band gap modification enabled C-TiO_2_ to harness visible light more efficiently, generating higher levels of ROS that facilitated biofilm disruption. Notably, C-TiO_2_ NPs displayed greater efficacy in eradicating biofilms of both Gram-positive and Gram-negative, with the latter typically more resistant to treatment due to its additional outer membrane barrier. The relatively positive surface charge of C-TiO_2_ further enhanced its interaction with the negatively charged bacterial cell walls contributing to its superior antibiofilm activity. This study provides valuable insights into the design of advanced photocatalytic materials for antimicrobial applications, demonstrating that carbon doping in TiO_2_ can yield more effective antibiofilm agents under visible light. Such materials offer a promising approach to addressing the challenges of biofilm-associated antibiotic resistance and environmental contamination. Our findings suggest that C-TiO_2_ NPs could serve as potent antimicrobial agents in various applications, paving the way for more sustainable and effective strategies to combat biofilm-related infections and improve environmental hygiene.

## Supplementary Material



## Notes

Absorbance spectra of various TiO_2_ NP samples; photographs of the different TiO_2_ samples; Tauc plots of the different TiO_2_ NP samples; ATR-FTIR spectra for the different TiO_2_ samples; TEM images of the annealed nanoparticles; atomic concentration derived from XPS survey scan for the different TiO_2_ samples; XPS survey scan of commercial TiO_2_ NP samples; HR-XPS of Cl *2p* scans of as-prepared and annealed C-TiO_2_ NP samples (PDF)
