# Supplementary material for: Tailored TiO2 Nanoparticles for Broad-Spectrum Antibiofilm Applications: A Systematic Comparison of Structural and Functional Properties of Carbon- and Nitrogen-Doped TiO2 Nanoparticles
Source: ACS Appl Eng Mater. 2026 Feb 3;4(2):882–96. doi: 10.1021/acsaenm.5c01089 (PMC12954750; doi:10.1021/acsaenm.5c01089)
Supplement: Supplementary file 1 [file em5c01089_si_001.pdf]

# Supporting Information

# Tailored $TiO_2$ Nanoparticles for Broad-Spectrum Antibiofilm Applications: A Systematic Comparison of Structural and Functional Properties of Carbon- and Nitrogen-Doped $TiO_2$ Nanoparticles

*Yu Hsin Tsai,[a#] Maheshika Kumarihamy,[a#] Nicole Beatrice Ponce,[a] Md. Masud Alam,[a] Wooram Kim,[a] Xiong Yu,[b] Tae Kyong John Kim,[c]* and Anna Cristina S. Samia[a,b]**

[a]Department of Chemistry, Case Western Reserve University, 10900 Euclid Avenue, Cleveland, Ohio 44106, United States

[b]Department of Civil and Environmental Engineering, Case Western Reserve University, 10900 Euclid Avenue, Cleveland, Ohio 44106, United States

[c]Swagelok Center for Surface Analysis of Materials, Case Western Reserve University, 10900 Euclid Avenue, Cleveland, Ohio 44106, United States

***Corresponding Authors:** Anna Cristina S. Samia, anna.samia@case.edu

Tae Kyong John Kim, tjkl55@case.edu

[#]Yu Hsin Tsai and Maheshika Kumarihamy equally contributed to this work

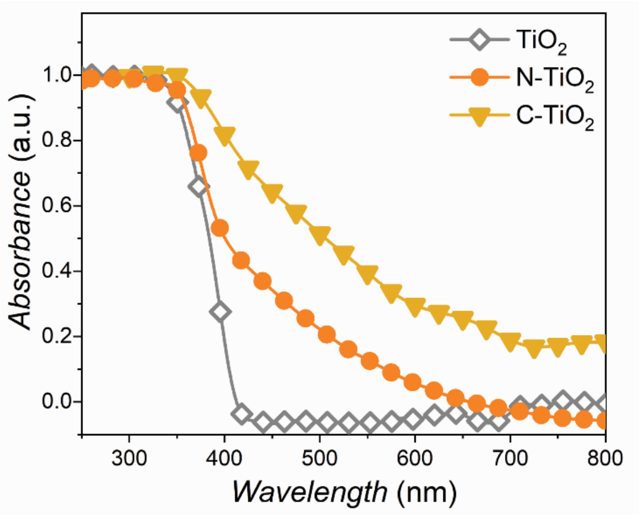


**Figure S1.** UV-vis absorbance spectra of the different $TiO_2$ NP samples, including commercial $TiO_2$ (control), N-$TiO_2$, and C-$TiO_2$ NPs.

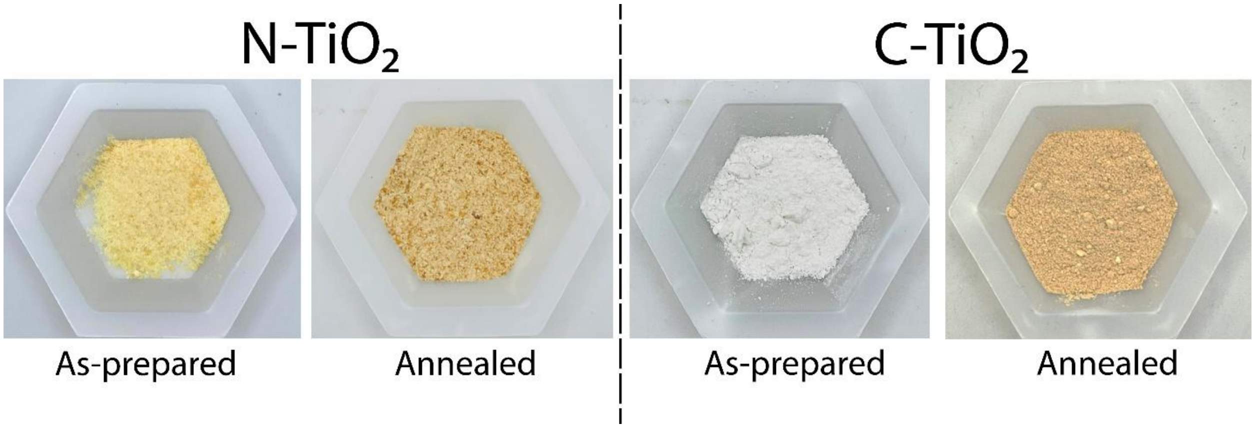


**Figure S2.** Photographs of the $TiO_2$ NP samples, including as-prepared and annealed N-$TiO_2$, and as-prepared and annealed C-$TiO_2$ NPs.

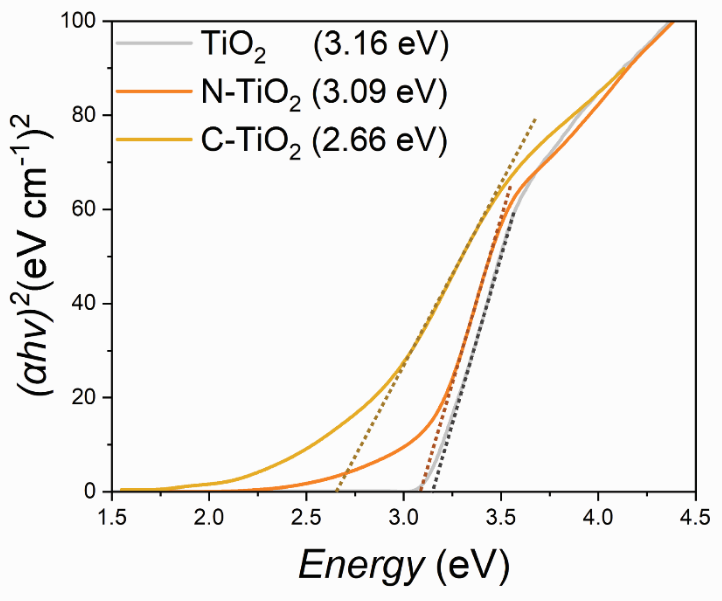


**Figure S3.** Tauc plots of the $TiO_2$ NP samples, including commercial $TiO_2$ (control), N-$TiO_2$, and C-$TiO_2$ NPs.

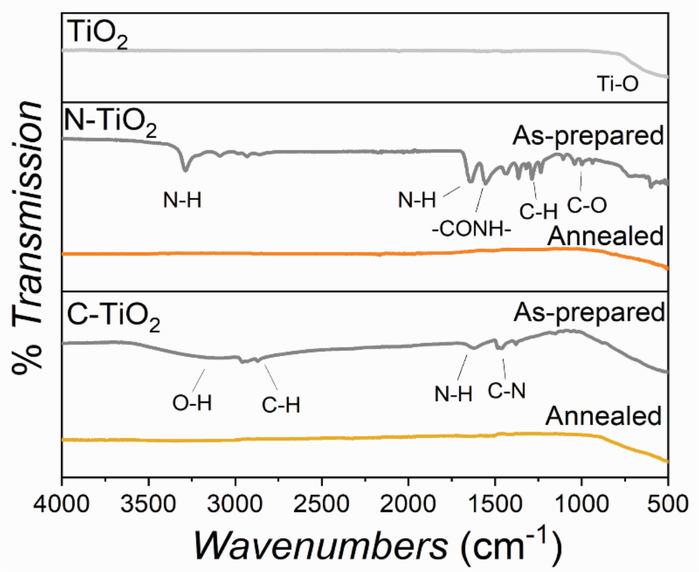


**Figure S4.** Attenuated total reflectance Fourier transform infrared (ATR-FTIR) spectra of the $TiO_2$ NP samples, including commercial $TiO_2$ (control), as-prepared and annealed N-$TiO_2$, and as-prepared and annealed C-$TiO_2$ NPs.

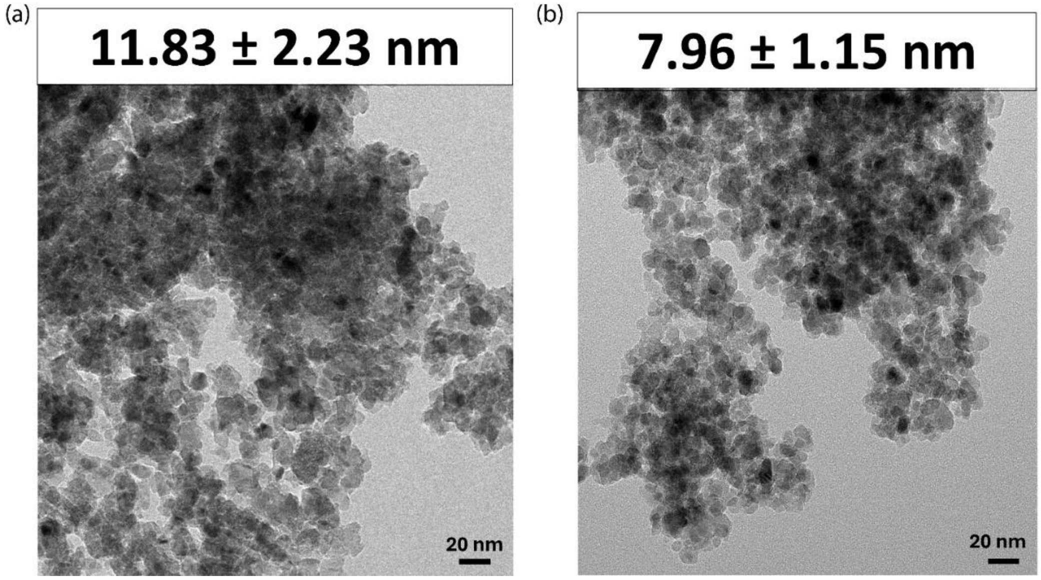


**Figure S5.** TEM images of the annealed non-metal doped $TiO_2$ NPs: (a) N-$TiO_2$ NPs and (b) C-$TiO_2$ NPs.

**Table S1.** Atomic composition derived from XPS survey spectral of the different $TiO_2$ NP samples, including as-prepared and annealed N-$TiO_2$, and as-prepared and annealed C-$TiO_2$ NPs.

| | N-$TiO_2$ | | C-$TiO_2$ | |
|---|---|---|---|---|
| Element | As-prepared | Annealed | As-prepared | Annealed |
| Ti | 6.1 ± 0.7% | 29.4 ± 1.1% | 15.7 ± 0.5% | 34.9 ± 1.0% |
| O | 17.3 ± 0.8% | 50.4 ± 1.4% | 35.7 ± 1.1% | 48.7 ± 1.1% |
| C | 57.7 ± 1.5% | 14.7 ± 1.5% | 41.2 ± 1.3% | 14.1 ± 2.2% |
| N | 18.9 ± 1.0% | 5.5 ± 0.3% | 0.0 ± 0.0% | 0.0 ± 0.0% |
| Cl | 0.0 ± 0.0% | 0.0 ± 0.0% | 7.4 ± 0.9% | 2.3 ± 0.3% |

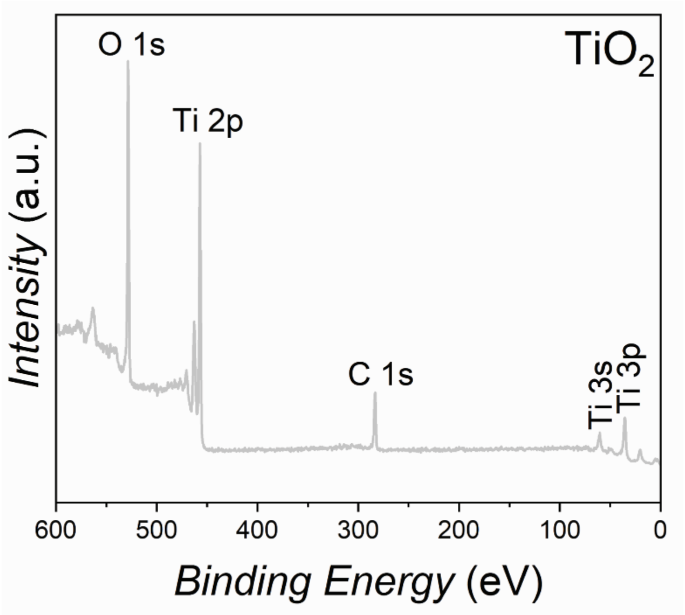


**Figure S6.** XPS survey spectrum of commercial $TiO_2$ NP sample.

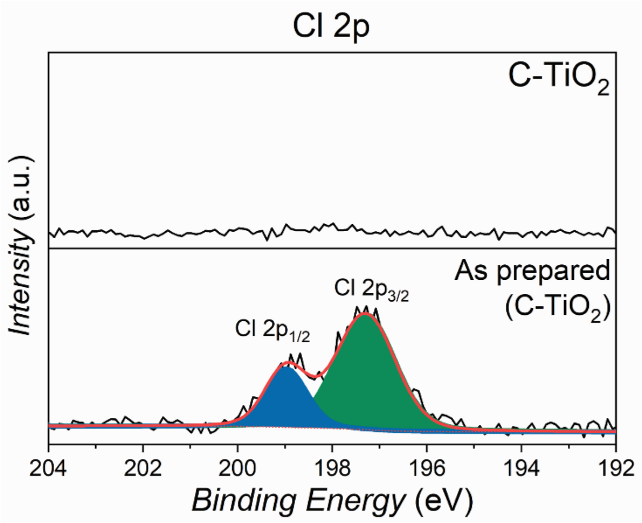


**Figure S7.** HR-XPS spectra of the Cl *2p* region obtained from the as-prepared and annealed C-$TiO_2$ NP samples.
